# FTO is required for myogenesis by positively regulating mTOR-PGC-1*α* pathway-mediated mitochondria biogenesis

**DOI:** 10.1038/cddis.2017.122

**Published:** 2017-03-23

**Authors:** Xiaobo Wang, Ning Huang, Min Yang, Dandan Wei, Haoran Tai, Xiaojuan Han, Hui Gong, Jiao Zhou, Jianqiong Qin, Xiawei Wei, Honghan Chen, Tingting Fang, Hengyi Xiao

**Affiliations:** 1 Lab for Aging Research, Center of Gerontology and Geriatrics, State Key Laboratory of Biotherapy, West China Hospital, Sichuan University and Collaborative Innovation Center, Chengdu 610041, China

## Abstract

Global germ line loss of fat mass- and obesity-associated (*FTO*) gene results in both the reduction of fat mass and lean mass in mice. The role of FTO in adipogenesis has been proposed, however, that in myogenesis has not. Skeletal muscle is the main component of body lean mass, so its connection with FTO physiologic significance need to be clarified. Here, we assessed the impact of FTO on murine skeletal muscle differentiation by *in vitro* and *in vivo* experiments. We found that FTO expression increased during myoblasts differentiation, while the silence of FTO inhibited the differentiation; in addition, skeletal muscle development was impaired in skeletal muscle FTO-deficient mice. Significantly, FTO-promoted myogenic differentiation was dependent on its m6A demethylase activity. Mechanically, we found that FTO downregulation suppressed mitochondria biogenesis and energy production, showing as the decreased mitochondria mass and mitochondrial DNA (mtDNA) content, the downregulated expression of mtDNA-encoding genes and peroxisome proliferator-activated receptor gamma coactivator 1 alpha (*PGC-1α*) gene, together with declined ATP level. Moreover, the involvement of mTOR-PGC-1*α* pathway in the connection between FTO and muscle differentiation is displayed, since the expression of FTO affected the activity of mTOR and rapamycin blocked FTO-induced PGC-1*α* transcription, along with the parallel alteration pattern of FTO expression and mTOR phosphorylation during myoblasts differentiation. Summarily, our findings provide the first evidence for the contribution of FTO for skeletal muscle differentiation and a new insight to study the physiologic significance of RNA methylation.

Fat mass- and obesity-associated (*FTO*) gene originally attracted attentions as an obesity- and diabetes-related protein owing to the significant association between its genetic polymorphism and BMI of human beings.^1, 2, 3, 4^ The linkage of FTO to body development and metabolic homeostasis is further demonstrated by experimental data. For instance, FTO overexpression leads to weight increase and its knockout induces weight loss in mice.^5, 6, 7^ It is clear now that FTO works as a nucleic acid demethylase capable of removing methyl groups from single-strand DNA and RNA,^8, 9, 10^ and therefore regulating *N*^6^-methyladenosine (m^6^A) level of RNA in cells.^11^ The cellular function of FTO remains largely unknown, as very limited biologic processes have been linked with the function of FTO. Given the predicted close association of FTO with energy homeostasis and body development, exploring the cellular processes connected with FTO function is a perspective research pursuit.

Studies have shown that FTO expression affects the hypothalamus-governed food intake in mice and the fat accumulation in different animals,^7, 12, 13, 14^ leading to the impression that FTO targets neural and adipose tissues and functions on central nervous system secretion and adipose differentiation. However, these findings cannot fully explain the data obtained from other experiments. For example, the homological knockout of the *FTO* gene caused >50% of mice death embryonically, and the reduction of body weight, not only the weight of fat mass but also that of lean mass.^7, 14^ In addition, FTO expression in non-neural and non-fat cells influenced the cell proliferation.^15^ Combining these data with the fact that FTO expresses in many mammalian tissues,^7^ it is reasonable to persuade the biologic function of FTO is not limited at neural and adipose tissues.

Skeletal muscle is the biggest energy-producing and -consuming organ in human body and also releases various myokines that participate in the metabolism regulation of the whole body.^16^ Accordingly, the disorders of skeletal muscle are involved in metabolic diseases.^17^ Differentiated and functional muscle cells are called myotubes, and derived from myoblasts during embryo and post-natal periods. Muscle maintenance and repair in adulthood are basically dependent on the differentiation of satellite cells, which are quiescent muscle precursors locating along with muscle fibers.^18^ Upon activation, satellite cells reenter the cell cycle to proliferate and differentiate to myoblasts, followed by the maturation of multinucleated muscle fibers.^19^ The process of muscle differentiation or myogenesis is controlled by various transcription factors, particularly by myogenic regulatory factors (MRFs). Among MRFs, MYF5 and MYOD are crucial for myogenic determination, while myogenin and MRF4 mainly drive the terminal differentiation.^20^ Although increasing knowledge have been achieved, the exact mechanism regarding to skeletal muscle differentiation has not been fully established, that is not an ideal state for developing new approaches to govern metabolic homeostasis and confront muscle-related disorders.

Energy supply is another critical factor for muscle differentiation.^21^ A series of studies proposed the role of mTOR-PGC-1*α*-mitochondria axis in the regulation of myogenesis. The facts are that the impairment of mitochondrial function blocks myogenic differentiation^22, 23^ and mTOR signaling pathway, a master regulator of cell metabolism and energy homeostasis,^24^ and positively regulates PGC-1*α* expression,^25^ a coactivator that controls mitochondrial biogenesis.^26^ Therefore, a situation capable of affecting the activity of mTOR pathway should be a potential regulator of mitochondria function and muscle differentiation.

The role of FTO in skeletal muscle is poorly understood, despite recently a couple of studies mentioned that the cells lacking FTO have decreased activity of the mTORC1 pathway,^15^ and the FTO-deficient mice have reduced lean mass.^6^ These observations suggest a potential connection among FTO, mTOR and myogenesis, raising a new hypothesis that FTO probably plays a role in muscle differentiation through mTOR-dependent manner.

Therefore, we asked if FTO can regulate myogenic differentiation in the present study and tried to elucidate the possible mechanism. For this sake, *in vitro* and *in vivo* experiments were performed using cultural myoblasts and skeletal muscle-specific FTO-deficient mice. Our results indicate the positive association between FTO protein and skeletal muscle differentiation. Furthermore, our results reveal that FTO regulates myogenic differentiation, at least in part through mTOR-PGC-1*α*-mitochondria axis. This is the first time to demonstrate the role of FTO in myogenic differentiation, and our study also provides a new line of evidence stressing the importance of mitochondria function on muscle differentiation.

## Results

### FTO expression influences myogenic differentiation *in vitro*

**Figure 1:**
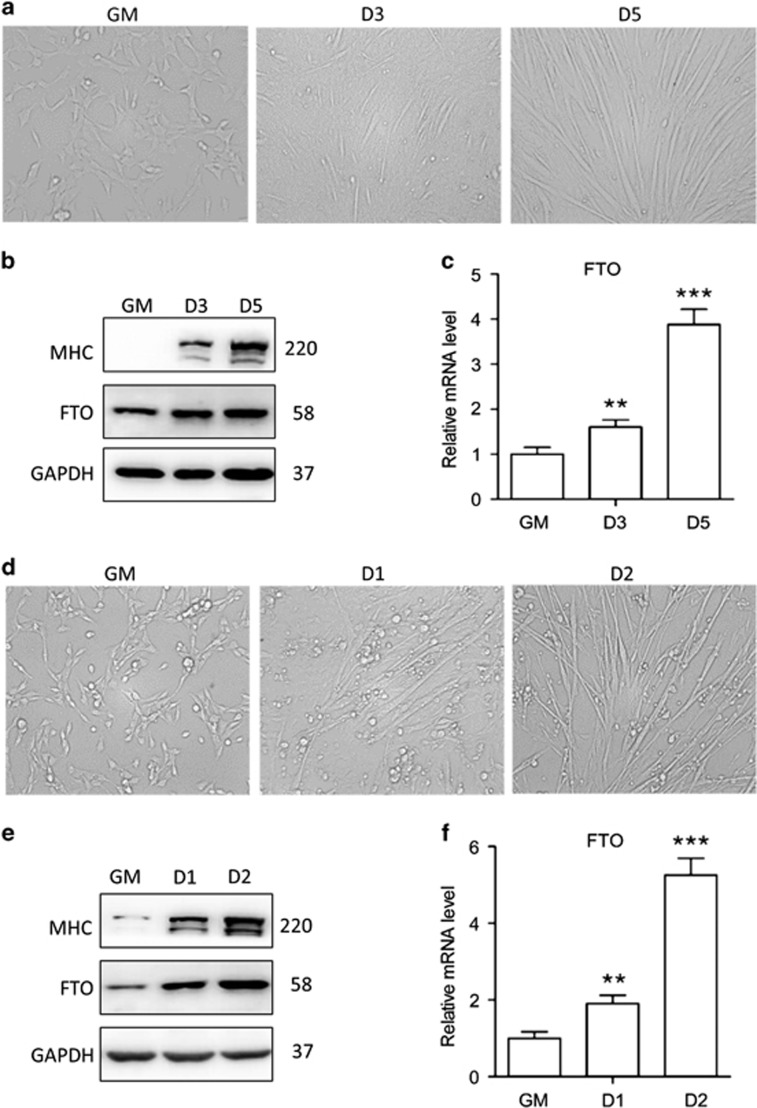
Endogenous FTO expression elevates during myogenic differentiation. C2C12 cells and MPM cells were differentiated for indicated days in DM, phase-contrast microscopy of differentiated C2C12 cells (**a**) and MPM cells (**d**). Western blot analysis of whole-cell lysates from differentiated C2C12 cells (**b**) and MPM cells (**e**) with indicated antibodies. qRT-PCR analysis of differentiated C2C12 cells (**c**) and MPM cells (**f**) with FTO. Asterisks indicate statistical significance (*P<0.05, **P<0.01, ***P<0.001)

As the first step for understanding the correlation between FTO expression and myogenic differentiation, we measured the endogenous FTO expression during the differentiation process of two cell models; both go from myoblasts to myotubes under novel cultivation condition described in Materials and Methods section. As shown, along with the differentiation of C2C12 myoblasts (Figure 1a), the protein level (Figure 1b) and the mRNA level (Figure 1c) of FTO both increased, similar to the situation observed in mouse primary myoblasts (MPMs) (Figures 1d–f). These results indicate that the expression of endogenous FTO is upregulated during myogenic differentiation.

**Figure 2:**
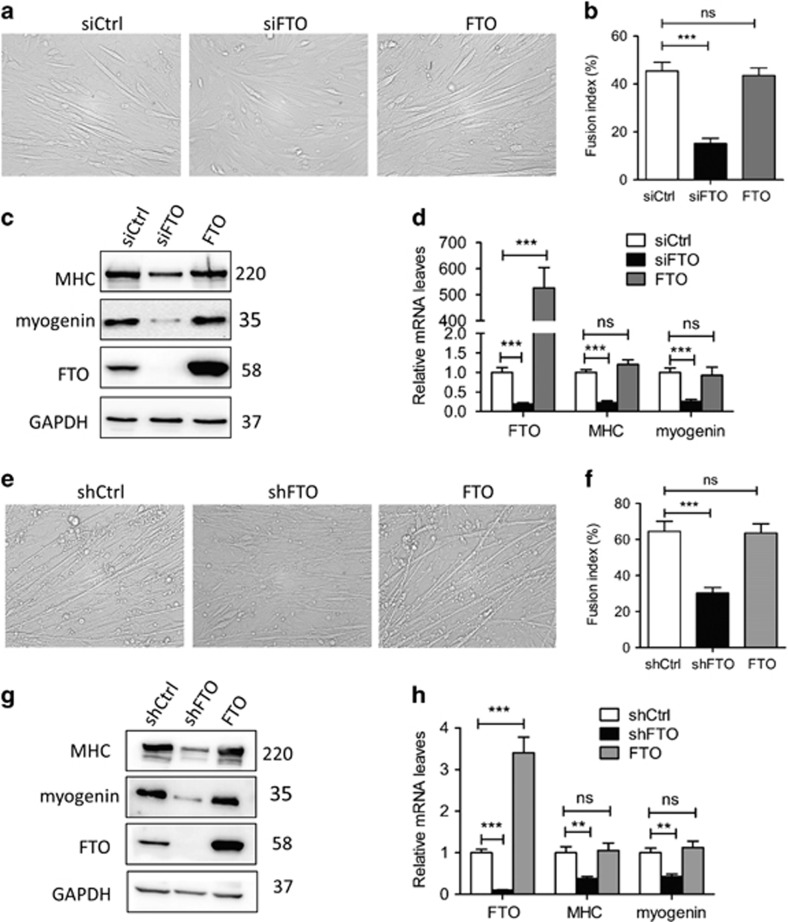
FTO expression influences myogenic differentiation. (**a–d**) C2C12 cells were transfected with FTO or control siRNA or FTO expression vector. Differentiation was induced for 3 days after 48 hours of transfection. (**a**) Phase-contrast microscopy of differentiated C2C12 cells. (**b**) Quantification of fusion index in (**a**). (**c**) Western blot analysis of whole-cell lysates from differentiated C2C12 cells with indicated antibodies. (**d**) qRT-PCR analysis of differentiated C2C12 cells with indicated genes. (**e–h**) MPM/shCtrl, MPM/shFTO and MPM/FTO cells were differentiated for 2 days, (**e**) phase-contrast microscopy of differentiated MPM cells. (**f**) Quantification of fusion index in (**e**). (**g**) Western blot analysis of whole-cell lysates from differentiated MPM cells with indicated antibodies. (**h**) qRT-PCR analysis of differentiated MPM cells with indicated genes. Asterisks indicate statistical significance (*P<0.05, **P<0.01, ***P<0.001)

Then, we silenced or overexpressed *fto* to see if the alteration of *fto* can influence myogenic differentiation. Silencing the *fto* by siRNA in C2C12 myoblasts, decreased the myotubes formation during differentiation (Figures 2a and b). Consistently, the protein level of myogenin and myosin heavy chain (MHC), early and late differentiation markers, respectively, decreased in FTO silenced C2C12 cells (Figure 2c), together with an apparent decrease of myogenin and MHC mRNA (Figure 2d). To strengthen these findings, we established two MPM pools with *fto* stably silenced or overexpressed by lentivirus, refer to as MPM/shFTO myoblasts and MPM/FTO myoblasts, respectively. We found the growth rate was around 70% and the protein level of cell cycle regulator Cyclin D1 was reduced in MPM/shFTO cells compared with MPM/shCtrl cells (Supplementary Figure 1). Therefore, we seeded more MPM/shFTO cells before differentiation induction to make the number of these cells equal with that of MPM/shCtrl cells when the differentiation was initiated. For MPM/shFTO myoblasts, the result was consistent with that seen in C2C12 cells with transfection of FTO siRNA, clearly showing that FTO silence suppressed myogenic differentiation. As to MPM/FTO myoblasts, however, the phenotype alteration was not observed, since the situation of myogenic differentiation was almost similar in FTO overexpressed cells and control cells (Figures 2e and f), resembled as observed when FTO overexpressed in C2C12 cells. Paralleling with the morphologic assessment, the influence of FTO expression on myogenic differentiation in these cells was confirmed by detecting myogenin and MHC expression (Figures 2g and h). These results suggest that FTO is required for myogenic differentiation.

### Skeletal muscle development is impaired in skeletal muscle FTO deficiency mice

**Figure 3:**
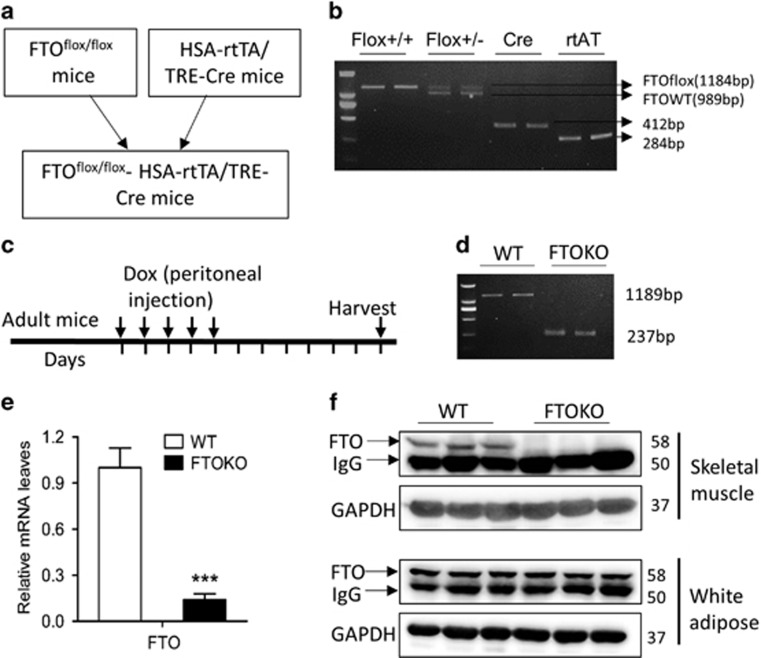
Generation of skeletal muscle FTO deficiency adult mice. (**a**) The schematic diagram of generation of skeletal muscle FTO deficiency mice. (**b**) PCR analysis of genomic DNA clearly discriminated the FTO^flox/flox^ and FTO^flox/^^−^ genotypes. (**c**) Doxycycline (Dox) regimen assay scheme. Vertical lines indicate daily intervals. PCR on genomic DNA (**d**) and qRT-PCR (**e**) of skeletal muscle from WT and FTOKO adult mice. (**f**) Western blot analysis of whole-cell lysates of rectus femoris and white adipose tissue from WT and FTOKO adult mice with indicated antibodies. Asterisks indicate statistical significance (*P<0.05, **P<0.01, ***P<0.001)

To gain *in vivo* evidence for the role of FTO in myogenesis, doxycycline-inducible skeletal muscle-specific FTO knockout mice were generated. The procedure includes three steps: (1) crossing FTO^flox/flox^ mice with doxycycline-inducible skeletal muscle-specific-expressed CRE mice (HSA-Cre mice) and getting FTO^flox/flox^ HSA-Cre mice (Figures 3a and b), (2) treating these mice with doxycycline (Figure 3c) to implement FTO knockout in skeletal muscle of adult mice and getting FTO^flox/flox^ (WT) and FTO^flox/flox^ HSA-Cre (FTOKO) mice, (3) measuring in genomic DNA demonstrates that exon 3 of the *FTO* gene was efficiently deleted in rectus femoris of FTOKO *versus* WT mice (Figure 3d), and examining the muscle specificity of FTO knockout by quantitative real-time PCR (qRT-PCR) and western blot assays (Figures 3e and f).

**Figure 4:**
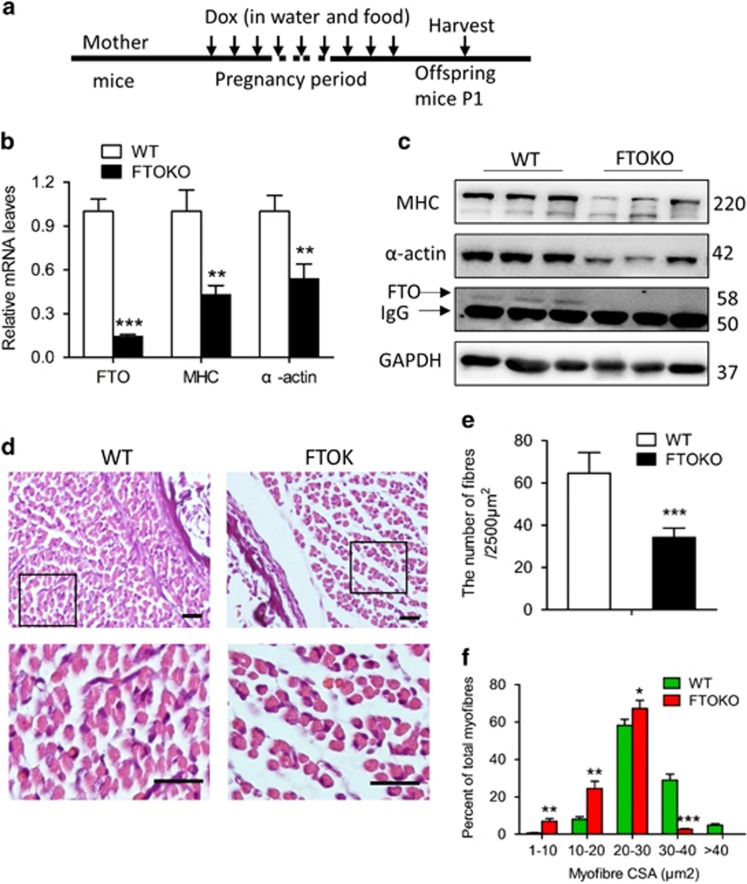
Skeletal muscle development is impaired in skeletal muscle FTO deficiency mice. (**a**) Dox regimen assay scheme. Vertical lines indicate daily intervals. (**b**) qRT-PCR and (**c**) western blot analysis of whole-cell lysates of hindlimb muscles from WT and FTOKO offspring P1 mice with indicated genes and antibodies, respectively. (**d**) H&E analysis of hindlimb muscles from WT and FTOKO offspring P1 mice. Scale bars: 20 *μ*m. (**e**) Quantification of H&E in (**d**). (**f**) Distributions of fiber sizes were analyzed. Asterisks indicate statistical significance (*P<0.05, **P<0.01, ***P<0.001)

By administrating doxycycline to pregnant FTO^flox/flox^ HSA-Cre mice crossed with FTO^flox/flox^ mice, we evaluated the role of FTO expression in the skeletal muscle development of their newborn offspring (Figure 4a). Then, we confirmed that FTO was depleted in hindlimb muscle of these newborn mice (Figures 4b and c). The body and hindlimb weight and the length of hindlimb of WT and FTOKO offspring P1 mice were no different (Supplementary Figures 2a and b). In line with our *in vitro* experiments, MHC and *α*-actin expression in the muscle of FTOKO mice was lower than that of WT littermates (Figures 4b and c). Furthermore, based on H&E-stained tissue section of hindlimb muscles, we found that the size of myofibers obviously reduced, with enlarged interval gap and decreased number of myofibers (Figures 4d and e, and Supplementary Figure 2c). By Image J program (NIH, Bethesda, MD, USA) analysis, it was found that, in comparison with the WT, the number of smaller sized myofibers <30 *μ*m^2^ in area were significantly more in FTOKO mice, whereas larger sized myofibers beyond 30 *μ*m^2^ in area were much less (Figure 4f). These results provided *in vivo* evidence for the influence of FTO expression on myogenesis.

### The demethylase activity of FTO is required for myogenic differentiation

**Figure 5:**
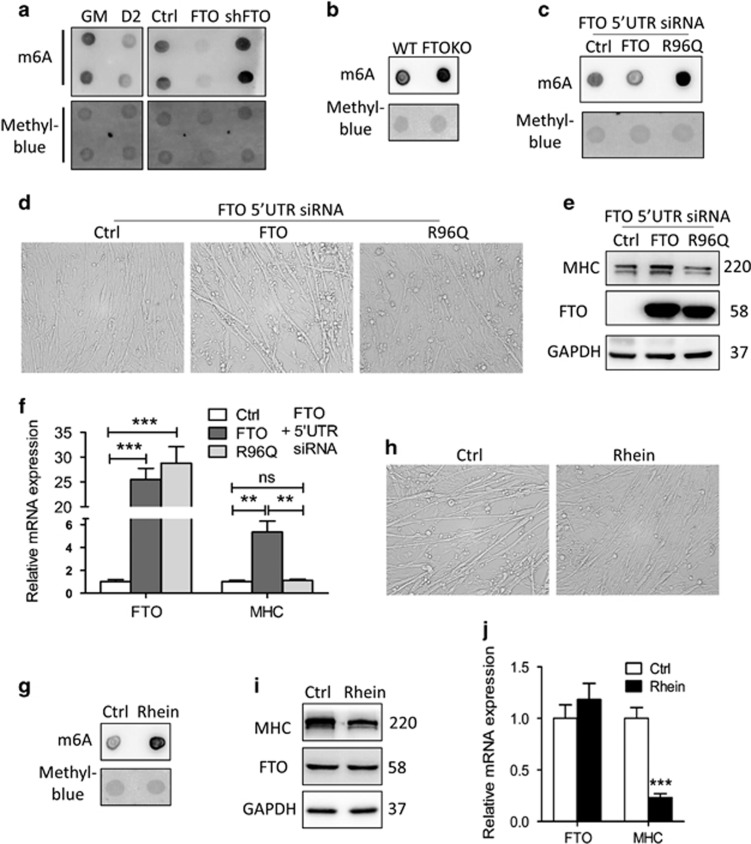
The RNA demethylase activity of FTO is required for myogenic differentiation. (**a**) mRNA was isolated from multiple stages (D0/2) of myogenesis and MPM/shCtrl, MPM/shFTO and MPM/FTO cells, and used in dot blot analyzes with m6A antibody. mRNA was loaded repeatedly. The m6A contents are shown in the upper panel. Equal loading of mRNA was verified by methylene blue staining (lower panel). (**b**) Dot blot analyses of m6A levels in mRNA of hindlimb muscles from WT and FTOKO offspring P1 mice. (**c**–**f**) MPM/shCtrl, MPM/FTO and MPM/R96Q cells were transfected with FTO mRNA 5′-UTR-specific siRNA, (**c**) dot blot analyses of m6A levels in mRNA. Differentiation was induced for 2 days, (**d**) phase-contrast microscopy of differentiated MPM cells. (**e**) Western blot analysis of whole-cell lysates from differentiated MPM cells with indicated antibodies. (**f**) qRT-PCR of differentiated MPM cells with indicated genes. (**g**–**j**) MPM cells were treated with rhein (10 *μ*g/ml) in GM for 2 days. (**g**) Dot blot analyses of m6A levels in mRNA. In DM for 2 days, (**h**) phase-contrast microscopy of differentiated MPM cells. (**i**) Western blot analysis of whole-cell lysates from differentiated MPM cells with indicated antibodies. (**j**) qRT-PCR of differentiated MPM cells with indicated genes. Asterisks indicate statistical significance (*P<0.05, **P<0.01, ***P<0.001)

Given FTO is a demethylase of *N*^6^-methyladenosine (m^6^A) in RNA,^11^ we next collected data to assess whether FTO-mediated m^6^A demethylation in mRNA is required for myogenesis. First, we measured the m^6^A level of mRNA by dot blot assay, and observed a severe decrease during myogenic differentiation and in MPM/FTO cells, whereas an obvious increase in MPM/shFTO cells and in the muscle of FTOKO newborn mice (Figures 5a and b). To ensure the responsibility of m^6^A dot blot assay for FTO activity, we generated MPM cells stably overexpressing wild-type FTO protein or a point mutation of FTO protein that lacks the demethylase activity of FTO (R96Q),^10^ and conducted a measure under the condition where endogenous FTO expression was repressed by FTO mRNA 5′-UTR-specific siRNA. As shown in Figure 5c, the m^6^A level of mRNA significantly increased in MPM/R96Q cells, compared with MPM/FTO cells. Parallel, R96Q overexpression impaired the differentiation of MPM cells (Figure 5d), as the protein and mRNA levels of MHC declined (Figures 5e and f). The final set of evidence came from the use of pharmaceutical compound rhein, a verified inhibitor of FTO demethylase.^27^ Similar with the effect of FTO silence, rhein treatment remarkably increased the m^6^A level of mRNA (Figure 5g) and impaired the differentiation of primary myoblasts (Figure 5h), with decreased MHC protein and mRNA level (Figures 5i and j). As expected, rhein did not affect the expression of FTO (Figures 5i and j). These results indicated that the m^6^A demethylation activity of FTO is required for its influence on myogenesis.

### FTO affects mitochondria biogenesis and function during myogenic differentiation

**Figure 6:**
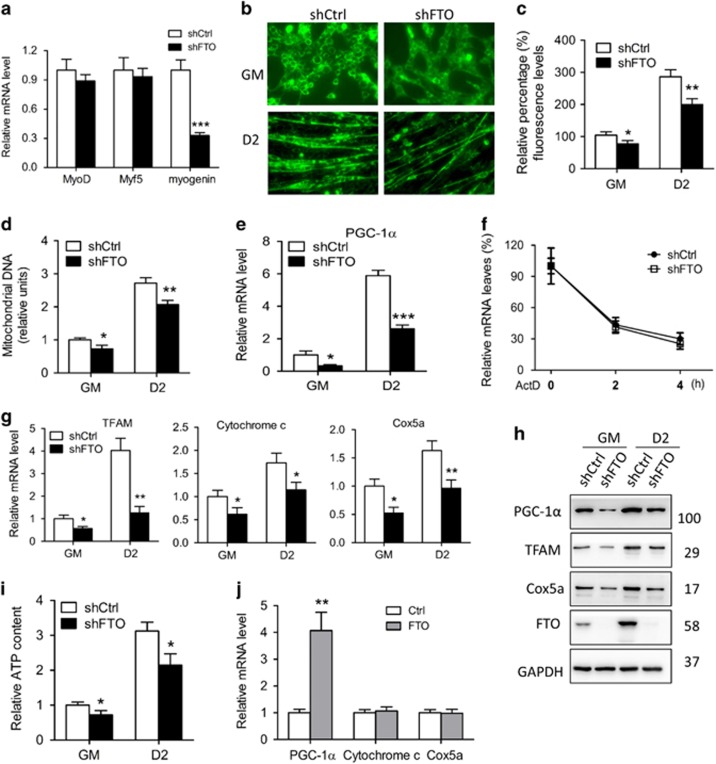
FTO affects mitochondria biogenesis and function during myogenic differentiation. (**a**) qRT-PCR of MPM/shctrl and MPM/shFto cells in GM with indicated genes and in DM for 2 days with myogenin. (**b**–**d**) Mitochondrial content as assessed by MitoTracker Green probe staining, (**b**) and quantified by fluorescence microplate reader (**c**) and mitochondrial DNA copy number (**d**) from multiple stages (D0/2) of myogenesis. (**e**) qRT-PCR and (**f**) mRNA stability of MPM/shCtrl and MPM/shFTO cells in GM with PGC-1*α*. (**g**) qRT-PCR of MPM/shCtrl and MPM/shFTO from multiple stages (D0/2) of myogenesis with indicated genes. (**h**) Western blot analysis of whole-cell lysates from MPM/shCtrl and MPM/shFTO cells from multiple stages (D0/2) of myogenesis with indicated antibodies. (**i**) ATP levels from multiple stages (D0/2) of myogenesis. (**j**) qRT-PCR of MPM/shCtrl and MPM/FTO cells from multiple stages (D0/2) of myogenesis with indicated genes. Asterisks indicate statistical significance (*P<0.05, **P<0.01, ***P<0.001)

To explore the mechanism underlying the role of FTO in myogenic differentiation, we measured the expression of several MRFs during the differentiation of myoblasts. Two well-known MRFs acting for the initiation of myogenesis, MyoD and Myf5, were not influenced upon FTO silence at mRNA level (Figure 6a). However, the mRNA of myogenin, encoding a MRF downstream of MyoD and Myf5, decreased in FTO silence cells on day 2 of differentiation (Figure 6a).

Considering mitochondria dysfunction is an important reason for the downregulation of *myogenin* gene but not the *MyoD* and *Myf5* genes,^28^ we assumed that FTO may affect myogenin expression and consequential myogenesis through regulating mitochondrial functions. To verify this hypothesis, we evaluated the status of mitochondria function, particularly from the view of mitochondria biogenesis and ATP production. As to evaluate mitochondria biogenesis, we measured the mass of mitochondria by MitoTracker Green staining, the content of mitochondrial DNA (mtDNA) by qRT-PCR and the expression of the genes important for mitochondria biogenesis by qRT-PCR analysis. Our data show that the mass of mitochondria decreased in FTO silenced cells (Figures 6b and c) and the mtDNA content increased with differentiation but decreased in FTO silence cells (Figure 6d). In terms of the gene expression, the mRNA of PGC-1*α*, a master transcriptional coactivator for mitochondria biogenesis, was significantly declined upon FTO silence (Figures 6e and h), without alteration in mRNA stability (Figure 6f), and three downstream targets of PGC-1*α*, TFAM, cytochrome c and cox5a; all increased during myogenesis, while decreased upon FTO silence (Figures 6g and h). In respect of ATP production, we found that intracellular ATP level increased during myogenesis, but decreased upon FTO silence (Figure 6i). Reversely, although FTO overexpression increased PGC-1*α* expression, the expression of cytochrome c and cox5a was not influenced (Figure 6j). These results together suggest that FTO silence inhibits myogenin expression during myogenic differentiation by downregulating the expression of *PGC-1α* gene, and then represses mitochondria biogenesis and function.

### mTOR-PGC-1*α* pathway activation is crucial for the role of FTO in myogenic differentiation

**Figure 7:**
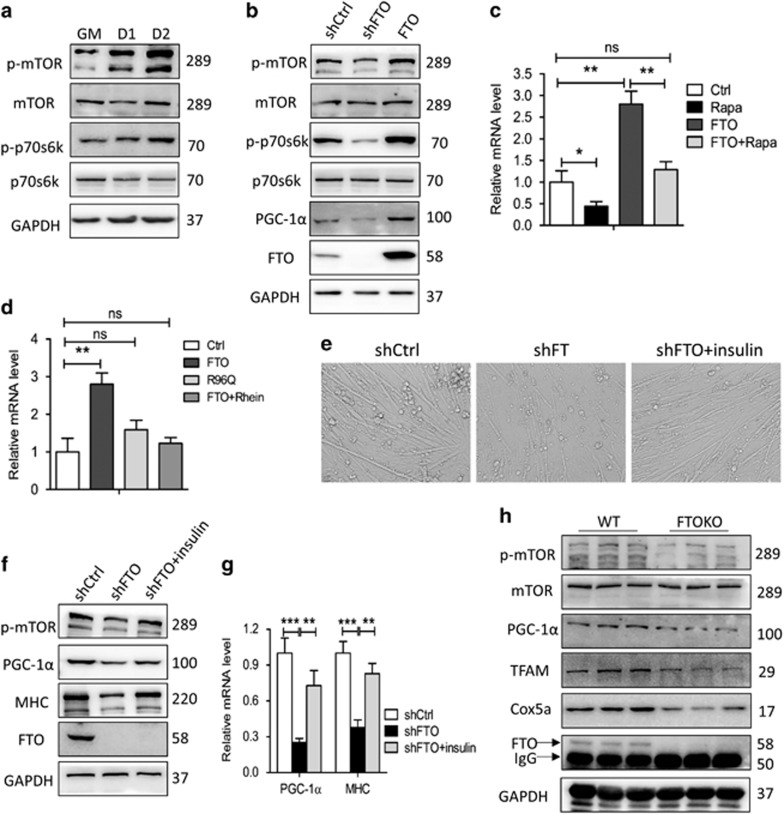
mTOR-PGC-1*α* pathway is crucial for the role of FTO in myogenic differentiation. (**a** and **b**) Western blot analysis of whole-cell lysates from differentiating MPM cells (**a**) and stably transfected MPM cells (**b**) with indicated antibodies. (**c** and **d**) qRT-PCR analysis of PGC-1α mRNA expression in original MPM cells, MPM/FTO cells or MPM/FTO R96Q cells; indicated groups were treated with rapamycin (100 nM, 16h) (**c**) or rhein (10 ug/ml, 48h) (**d**). (**e**–**g**) MPM/shFTO cells were treated with insulin (100 nM) in DM for 2 days, (**e**) phase-contrast microscopy of differentiated MPM cells. (**f**) Western blot analysis of whole-cell lysates from differentiated MPM cells with indicated antibodies. (**g**) qRT-PCR of differentiated MPM cells with indicated genes. (**h**) Western blot analysis of whole-cell lysates of hindlimb muscles from WT and FTOKO offspring P1 mice with indicated antibodies. Asterisks indicate statistical significance (*P<0.05, **P<0.01, ***P<0.001)

Since mTOR-PCG-1*α* controls mitochondria biogenesis^25^ and FTO can activate mTORC1,^15^ we asked whether FTO regulated mitochondrial biogenesis through mTOR-PGC-1*α* pathway. Western blotting assays confirmed that mTORC1 pathway was activated during myogenic differentiation (Figure 7a), and mTORC1 pathway together with PGC-1*α* expression was downregulated upon FTO silence (Figure 7b). In addition, PGC-1*α* mRNA level was higher in undifferentiated MPM/FTO cells and this increase was abrogated when treated MPM/FTO cells with mTOR inhibitor rapamycin (Figure 7c). Because of its ability in influencing myogenic differentiation, we tested whether FTO activity affects the level of PGC-1*α* mRNA. Interestingly, R96Q mutation of FTO protein and the rhein treatment resulted in similar alteration in PGC1a expression as that caused by rapamycin treatment (Figure 7d). In contrast, the treatment of cells with insulin, the mTOR activator, restored the decrease of myotubes (Figure 7e) and the protein and mRNA levels of MHC and PGC-1*α* (Figures 7f and g) in FTO silence cells. Consistently, PGC-1*α*, TFAM, Cox5a and the phosphorylation of mTOR (S2448) were also decreased in mouse muscles lacking FTO (Figure 7h). Together, our findings suggest that FTO acts on the upstream of mTOR-PGC-1*α* pathway to regulate myogenic differentiation.

## Discussion

In this study, we first demonstrated that FTO plays an important role in myogenic differentiation and skeletal muscle development, and its m6A demethylation activity is required for this role. We also found that the effect of FTO on muscle differentiation is mediated at least partially by mTOR-PGC-1*α*-mitochondria axis.

Several studies have shown FTO deficiency resulted in high perinatal lethality, and reduced body length, fat mass and lean mass in mice,^6, 7^ implying that FTO fundamentally impacts on the development and functions of body composition. Yun-gui Yang *et al.* reported that FTO regulates adipogenesis, and thereby influences fat mass and body weight.^29, 30^ We investigated the role of FTO in myogenesis, considering that skeletal muscle is a major component of lean mass and an essential insulin-sensitive organ similar as adipose.^31^ Our study not only revealed the contribution of FTO on myogenic differentiation in cultivated myoblasts, either established C2C12 myoblasts cell line or primary murine myoblasts, but also confirmed the impact of FTO on skeletal muscle development, using skeletal muscle-specific FTO-deficient mice. The *in vivo* evidence is novel as it was produced from mice with muscle-specific FTO depletion. This line of mice provides us convenience for evaluating the role of local FTO in myogenesis and muscle differentiation. In terms of this point, it is a good model and tool for *in vivo* study, because the complicated situation caused by the influence of FTO expressed in other tissues can be avoided. Given skeletal muscle is the key component of lean mass, our findings support the opinion that FTO influences lean mass development.

Many insights for the mechanism of muscle differentiation have been achieved, revealing that sequential expression of different MRFs is crucial.^20^ We particularly concerned the effect of FTO on myogenin, as myogenin is the key MRF that directly activates genes encoding myofiber proteins.^32^ Hinted by a previous study,^28^ we determined the connection of mitochondrial biogenesis and function with FTO-mediated myogenesis and myogenin expression. This result is understandable as the energy produced by mitochondria is indispensable for cell growth, differentiation and organic development,^33^ not to mention the differentiation and functioning of skeletal muscle requires particularly lots of energy supply.^34^

Then, how does FTO affect mitochondria biogenesis? Our data draft a cascade from FTO to mTOR and to PGC-1*α*, the master factor for mitochondria biogenesis. It has been reported that mTOR-YY1-PGC-1*α*-mitochondria axis exists in cells, where mTOR controls mitochondrial biogenesis and function through regulating the stability of YY1-PGC-1*α* transcriptional complex.^25^ This axis is partially confirmed in our study, showing that FTO influences the expression of PGC-1*α* and mitochondrial biogenesis with mTOR dependency. Moreover, although just being the preliminary information, our data indicates that FTO is an upstream regulator of mTOR pathway, coinciding with a finding published previously standing on the fact that FTO affects mTORC1 activity.^15^

FTO is a nucleic acid demethylase that removes methyl groups from both DNA and RNA.^8, 9, 10^ It is commonly accepted that its most important functional role is demethylation of *N*^6^-methyladenosine (m^6^A) in mRNA.^11^ For example, FTO controls exon splicing of adipogenic regulatory factor RUNX1T1 by regulating m^6^A levels around splice sites and thereby modulates differentiation.^29^ As FTO inhibitor rhein and FTO mutant (R96Q) that lacks demethylase activity inhibited myogenic differentiation, we consider the activity of FTO is required for myogenic differentiation. This finding provides new evidences and ideas for the mechanism study of skeletal muscle differentiation.

An interesting result in our study is that FTO depletion interferes with myogenic differentiation, while overexpression of FTO could not promote myogenic differentiation *in vitro*. The unmatched results from loss-of-function and strength-of-function experiments we got *in vitro* seem coincident with those reported previously. For example, according to the statement from Gao *et al.*,^7^ FTO deficiency in mice results in an obvious reduction of lean mass. However, in the demonstration of Church *et al.*,^5^ overexpression of FTO does not increase lean mass, at least in male mice. Although more data are needed, we would like to propose a brief explanation for these unmatched resultants based on the line of our finding. It is that FTO deficiency can inhibit myogenic differentiation because of its significant role in suppressing mitochondria biogenesis, which can severely interfere muscle differentiation requiring ATP consuming; on the other hand, the overexpression of FTO does not invoke marked change in differentiation, which may be owing to the enough capacity of endogenous FTO expression for supporting muscle differentiation. This notion should be strengthened by our data that the expression of Cytochrome *c* and *Cox5a* genes, directly participating in mitochondria biogenesis, does not change in FTO overexpression cells. It also remains that although being an important transcriptional coactivator involving in mitochondria biogenesis, PGC-1*α* expression level is not the only determinant of mitochondria biogenesis, so its expression could not lineally affect myogenic differentiation. Actually, many studies have confirmed that the function of PGC-1*α* can be regulated at protein modification level, for instance, it can be acetylated by GCN5 acetyltransferase complex,^35^ and phosphorylated by p38 MAP kinase, AMP kinase and Akt/PKB.^36, 37, 38^

Although we displayed that FTO could regulate myogenic differentiation, the precise mechanism of this relationship has not been revealed. It is still unclear how FTO affects the activity of mTOR pathway, which is the direct target gene of FTO as a demethylase of m^6^A of RNA. Further studies focused on these issues are needed.

In summary, our findings demonstrate that the function of FTO is required for myogenic differentiation and suggest FTO-mediated mTOR-PGC-1*α*-mitochondrial axis involved in this regulation. This study is a basic line for further investigation of the molecular mechanisms in the role of FTO during myogenic differentiation, and will be informative for developing our understanding about muscle differentiation, RNA methylation and mTOR pathway regulation.

## Materials and Methods

### Isolation of MPMs

Primary myoblasts from about 10-day-old C57BL/6J were isolated and cultured following the protocol of Gharaibeh *et al.*^39^ In brief, hindlimb muscles were minced mechanically and digested with enzyme mixture: 0.2% collagenase II (Invitrogen, cat. 17101015, Carlsbad, CA, USA) and 0.05% trypsin in DMEM (Gibco, Carlsbad, CA, USA) for 45 min at 37 °C with slight agitation. The tissue was triturated vigorously using 1 ml tip and passed through a 70 *μ*m filter, and the cells were collected by centrifugation. Cells were suspended in primary myoblasts growth media (DMEM supplemented with 20% FBS and 1% penicillin/streptomycin) for 2 h at 37 °C; the non-adherent cells were then transferred to another plate. To get pure satellite cells, the following procedure was performed in strict accordance with the purifying method in the study by Gharaibeh *et al.*^39^ After about 1 week, the satellite cells proliferate as myoblasts naturally.

### Cell culture

C2C12 and MPM were maintained at 37 °C and 5% CO_2_ in growth media (GM: DMEM medium containing 10% (v/v) FBS and 1% antibiotics). To induce myogenic differentiation, cells were grown to 70–80% confluence in GM and then switched to differentiation medium (DM: DMEM supplemented with 2% (v/v) horse serum and 1% antibiotics). A total of 293FT cells were also cultured in GM.

### Lentivirus packaging and cell lines

The vectors containing cDNA of wild-type FTO and R96Q mutant of FTO were kindly gifted by Renbin Zhao.^40^ Primers bearing Xba1 and Not1 sites were used to generate PCR fragments that were subcloned into pLVX-IRES-ZsPuro lentiviral expression vector (Clontech, Shiga, Japan). Lentiviral shRNA construct for mouse *FTO* gene was purchased from GENECHEN (Shanghai, China). The target sequence was: AGAACCATACTATTTGCTT. Lentivirus was produced by co-transfection of lentivirus packing plasmids with psPAX2 and pMD2.G using Jet PRIME (PolyPlus, Illkirch, France) into 293FT cells following manufacturer's instruction. Medium was changed 24 h post transfection and the medium containing virus was collected after 72 h, followed by a centrifugation at 10 000 g for 10 min. The supernatant was used to infect MPM cells in the presence of 10 *μ*g/ml polybrene (Sigma-Aldrich, cat. H9268, Carlsbad, CA, USA), or stored at −80 °C. Selection of resistant colonies was initiated 48 h later using 3 *μ*g/ml puromycin (Life Technology, Carlsbad, CA, USA; cat. A1113803).

### RNA interference

All siRNAs were purchased from Sheng Gong (Shanghai, China), the sequences used are provided in Supplementary Information. For knockdown experiments, cells were transfected with siRNAs using Jet PRIME (PolyPlus) following manufacturer's instruction. The transfection media were then replaced with DMEM supplemented with fetal bovine serum, and cells were ready for subsequent differentiation induction.

### Western blots

Cells were lysed in RIPA buffer with a cocktail of protease inhibitors (Biotool, cat. B14002, Houston, TX, USA). Twenty micrograms of protein extracts were separated by SDS-PAGE and transferred to PVDF membranes (Millipore, cat. GVWP2932A, Billerica, MA, USA). Membranes were blocked with 5% non-fat milk, followed by overnight incubation with primary antibodies against FTO (Abcam, cat. ab92821, Cambridge, UK), myogenin (Zen Bioscience, cat. 600252, Chengdu, China), MHC (eBioscience, cat. 14-6503, Carlsbad, CA, USA), GAPDH (Abcam, cat. ab181602), *α*-actin (Beyotime Biotechnology, cat. AA132, Beijing, China), p-mTOR (Cell Signaling Technology, cat. 5536, Danvers, MA, USA), mTOR (Cell Signaling Technology, cat. 2972), p-p70S6k (Cell Signaling Technology, cat. 9205), p70S6k (Cell Signaling Technology, cat. 9202), Cyclin D1 (Cell Signaling Technology, cat. 2922), PGC-1*α* (Sheng Gong, cat. D162041), TFAM (Zen Bioscience, cat. 600252) and Cox5a (Sheng Gong, cat. D261450). Detection was made with HRP-conjugated secondary antibody (Zen Bioscience, cat. 501926) and signals were detected with ECL Plus Western Blotting Reagent Pack (Bio-Red, Hercules, CA, USA).

### qRT-PCR analysis

Cells and skeletal muscle were collected and washed twice with PBS, and total RNA was extracted using Trizol reagent (Takara, Shiga, Japan) following the manufacturer's instructions. Reverse transcription for mRNA was carried out using cDNA Synthesis Super Mix (Biotool, cat. B24403). qRT-PCR was carried out in an ABI cycler using SYBR Green qPCR Master Mix (Biotool, cat. B21203), and the relative amount of cDNA was calculated by the comparative CT method using the 18S ribosomal RNA sequences as control. The primer sequences used are provided in Supplementary Information.

### Analysis of m6A levels in mRNA using dot blot

Cellular mRNA was isolated with Poly (A) Purist Kit (Biotool, cat. AM1916), and the concentration and quality of mRNA was determined by Nano-Drop (Thermo, Carlsbad, CA, USA). Purified mRNA was denatured at 95 °C for 5 min and cooled down on ice. Samples (150 ng) were spotted on Amersham Hybond-N+ membranes (GE Healthcare, cat. RPN303B, Chicago, IL, USA) and air dried for 5 min, then UV-crosslinked (2 × auto-crosslink, 1800 UV Stratalinker, STRATAGENE, La Jolla, CA, USA). Membranes were blocked with 5% non-fat milk in TBST for 1 h, and incubated with anti-m^6^A antibody (Abcam, cat. 151230) overnight at 4 °C. After three washes, membranes were incubated with HRP-linked secondary anti-rabbit IgG antibody for 1 h at room temperature. Signals were detected with ECL Plus Western Blotting Reagent Pack (Bio-Red).

### mRNA stability measurement

Cells were treated with actinomycin D (5 *μ*g/ml, MCE, cat. HY-17559, Princeton, NJ, USA) for 4, 2 and 0 h before collection. Total RNA was isolated by Trizol reagent. After reverse transcription, the mRNA levels of transcripts of interest were detected by qRT-PCR.

### Mitochondrial content assay

Mitochondrial content assays were carried out according to Iwabu *et al.*,^41^ with slight modifications. For quantification of mitochondrial content, we used mtDNA and MitoTracker Green probe (Life Technology).

mtDNA was quantified via qRT-PCR by measuring the ratio of mitochondrial-encoded *Cox2* gene to an intron of the nuclear-encoded *β-globin* gene. Primer sequences are provided in Supplementary Information.

MitoTracker Green probe preferentially accumulates in mitochondria regardless of the mitochondrial membrane potential and provides an accurate assessment of mitochondrial mass. Cells were washed with PBS and incubated at 37 °C for 30 min with 100 nM MitoTracker Green FM (Molecular Probes, Carlsbad, CA, USA). Cells were collected by using trypsin/EDTA and resuspended in PBS. Fluorescence intensity was detected with excitation and emission wavelengths of 490 and 516 nm, respectively, and the values were corrected for total protein (mg/ml).

### ATP level quantitation

Intracellular ATP level assay was performed using an ATP assay kit (Nanjing Jiancheng Biotechnology Institute, Nanjing, China) following the manufacturer's protocol. Samples were compared to ATP standards and then normalized to protein content by performing BCA method (Cwbio, Beijing, China).

### Generation of skeletal muscle FTO knockout mice

To generate doxycycline-inducible skeletal muscle-specific FTO deletion mice, FTO^flox/flox^ mice^14^ were crossed with HSA-Cre mice^42^ to generate FTO^flox/+^ HSA-Cre mice, which were then crossed to FTO^flox/flox^ mice to generate FTO^flox/flox^ and FTO^flox/flox^ HSA-Cre mice. Genotyping of the mice was performed by using PCR. Two primers were used for FTO and floxed FTO genotyping: 5′-AGCGCTCACTGGAGAGTGTCTG-3′, and the reverse primer was 5′-GAGCCAGAGAGGATTTAGATGGG-3′. Primers used for detection of Cre and HAS were 5′-AGGTGTAGAGAAGGCACTTA-3′, 5′-CTAATCGCCATCTTCCAGCA-3′, 5′-ACTGAGAGGTGGGAAGCTCA-3′ and 5′-GGCGAGTTTACGGGTTGTTA-3′. The sizes of the amplified products are 412 bp for Cre and 284 bp for HSA, respectively. All procedures involving animals were performed in conformity with relevant guidelines and regulations, and approved by the Ethics Committee of Sichuan University.

### Doxycycline administration

Doxycycline, a derivative of tetracycline, is a good choice for the induction of the Tet ON system. Mice of all genotypes were treated with doxycycline. To knock out FTO in mice, two methods were used to deliver doxycycline (MCE, cat. HY-N0565B). The pregnant females were given doxycycline-containing food (400 mg of doxycycline was dissolved in 50 ml of drinking water containing 10% sucrose and then mixed with 100 g of mouse chow) and water (containing 2 mg/ml of doxycycline and 5% sucrose) for the whole pregnancy period. The doxycycline–sucrose solution was administered *ad libitum* from foil-covered water bottles and prepared fresh every 3 to 4 days.

For adult male mice, doxycycline was freshly dissolved in 0.9% NaCl at a final concentration at 10 mg/ml and filter-sterilized prior to intraperitoneal injection at a dose of 35 mg/kg per day body weight for 5 days.

### Histological analysis

The hindlimb muscles from WT and FTOKO offspring P1 mice were collected. Muscles were preserved in 4% formaldehyde, bisected at the mid belly and embedded in paraffin perpendicularly with the same polarity. Then, H&E-stained cross sections from hindlimb muscles of each animal were reviewed. The numbers of the same area fibers (2500 *μ*m^2^) in five fields from each animal (*n*=5) were counted using the Image J program. The cross sectional area of each fiber (*n*>1000) in five fields from each animal (*n*=5) was determined using the Image J program.

### Statistical analysis

All experiments were conducted on at least three biological replicates. All values are presented as mean±S.E.M. Statistical analysis was performed using the GraphPad Prism(version 5.0) (GraphPad software, San Diego, CA, USA) using Student *t*-test. Asterisks indicate statistical significance (*P<0.05, **P<0.01, ***P<0.001).
