# Supplementary material for: FTO is required for myogenesis by positively regulating mTOR-PGC-1α pathway-mediated mitochondria biogenesis
Source: Cell Death Dis. 2017 Mar 23;8(3):e2702–. doi: 10.1038/cddis.2017.122 (PMC5386528; doi:10.1038/cddis.2017.122)
Supplement: Supplementary Information [file cddis2017122x1.doc]

Supplementary Figure 1


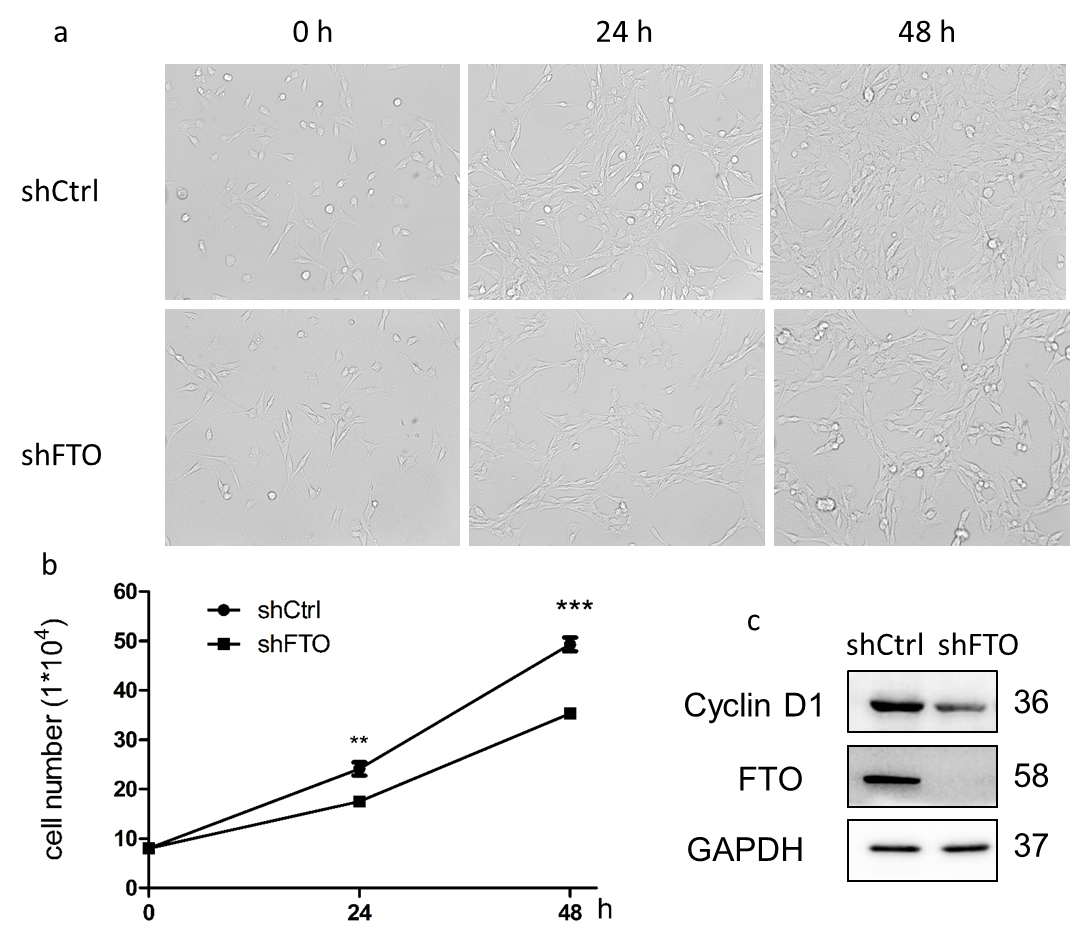


**Supplementary Fig. 1.** **FTO depletion reduced rates of cell growth.** For measurement of growth rate, MPM/shCtrl and MPM/shFTO were allowed to grow for different time periods. (a) Phase contrast microscopy and (b) the number of MPM/shCtrl and MPM/shFTO at different time periods. (c) Western blot analysis of whole cell lysates from MPM with indicated antibodies.

Supplementary Figure 2


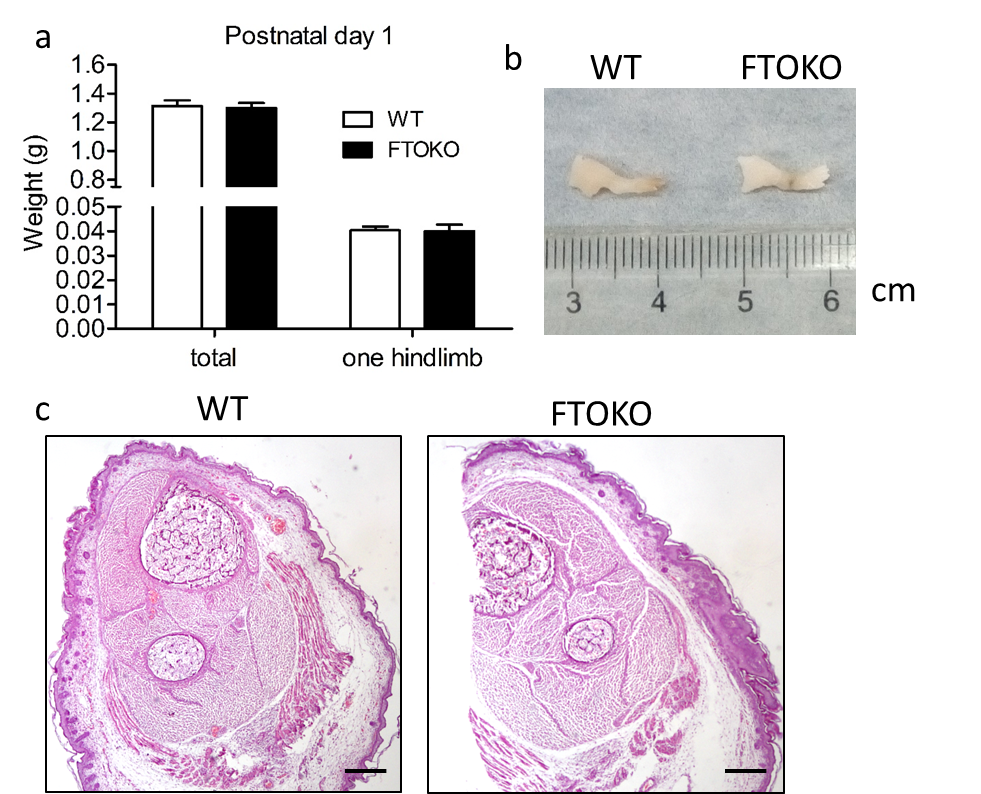


**Supplementary Fig. 2.** (a)Body and hindlimb weight of WT and FTOKO offspring P1 mice. (b) The length of hindlimb of WT and FTOKO offspring P1 mice. (c) H&E analysis of the cross section of hindlimb muscles from WT and FTOKO offspring P1 mice. Scale bars: 250 μm.

| **Gene Expression primers** |
| --- |

Myogenin-F: CCTGGAAGAAAAGGGACTGG

Myogenin-R: CGCTCAATGTACTGGATGGC

MHC-F: CTCAAGCTGCTCAGCAATCTATTT

MHC-R: GGAGCGCAAGTTTGTCATAAGT

18S-F: TTGACGGAAGGGCACCACCAG

18S-R: GCACCACCACCCACGGAATCG

Myf5-F: CACCACCAACCCTAACCAGAG

Myf5-R: AGGCTGTAATAGTTCTCCACCTG

MyoD-F: CCACTCCGGGACATAGACTTG

MyoD-R: AAAAGCGCAGGTCTGGTGAG

PGC-1α-F: ACTACAGACACCGCACACACC

PGC-1α-R: CCTTTCGTGCTCATAGGCTTC

TFAM-F: ATTCCGAAGTGTTTTTCCAGCA

TFAM-R: TCTGAAAGTTTTGCATCTGGGT

FTO-F: TTCATGCTGGATGACCTCAATG

FTO-R: GCCAACTGACAGCGTTCTAAG

Cox5a-F: GGGTCACACGAGACAGATGA

Cox5a-R: GGAACCAGATCATAGCCAACA

Cytochrome c-F: GGAGGCAAGCATAAGACTGG

Cytochrome c-R: TCCATCAGGGTATCCTCTCC

α-actin-F: CCCAAAGCTAACCGGGAGAAG

α-actin-R: CCAGAATCCAACACGATGCC

mtDNA primers:

Cox2-F: GCCGACTAAATCAAGCAACA

Cox2-R: CAATGGGCATAAAGCTATGG

β-globin-F: GAAGCGATTCTAGGGAGCAG

β-globin-R: GGAGCAGCGATTCTGAGTAGA

RNAi sequence

siCtrl: UUCUCCGAACGUGUCACGU

siFTO-1: GGUGCUCCGUGAAGUUAAA

siFTO-2: UUAAGGUCCACUUCAUCAUCGCAGG
